# Association of serum leptin and adiponectin with anthropomorphic indices of obesity, blood lipids and insulin resistance in a Sub-Saharan African population

**DOI:** 10.1186/s12944-016-0264-x

**Published:** 2016-05-17

**Authors:** Clarisse Noël A. Ayina, Jean Jacques N. Noubiap, Laurent Serge Etoundi Ngoa, Philippe Boudou, Jean François Gautier, Michel Karngong Mengnjo, Jean Claude Mbanya, Eugene Sobngwi

**Affiliations:** Department of Animal Science, Faculty of Science, University of Douala, Douala, Cameroon; Department of Medicine, Groote Schuur Hospital and University of Cape Town, Cape Town, South Africa; Medical Diagnostic Center, Yaounde, Cameroon; Department of Animal Science, Higher Teacher’s Training College, University of Yaoundé I, Yaounde, Cameroon; Department of Hormonal Biology, Saint-Louis Hospital, Public Assistance - Paris Hospitals, University Paris-Diderot Paris-7, Paris, France; Department of Diabetes and Endocrinology, Lariboisiere Hospital, Public Assistance - Paris Hospitals, University Paris-Diderot Paris-7, Paris, France; INSERM UMRS 1138, Cordeliers Research Centre, University Pierre et Marie Curie-Paris 6, Paris, France; Department of Internal Medicine and Specialties, Faculty of Medicine and Biomedical Science, University of Yaoundé I, Yaounde, Cameroon; Laboratory for Molecular Medicine and Metabolism, Biotechnology Center, University of Yaoundé I, Yaounde, Cameroon; National Obesity Center, Yaoundé Central Hospital, Yaounde, Cameroon

**Keywords:** Adipokines, Adiponectin, Leptin, Blood lipids, Insulin resistance, Insulin sensitivity, Obesity indices

## Abstract

**Background:**

There is little data on the metabolic effects of adipokines in sub-Saharan African populations. This study aimed to explore the potential relationship of leptin and adiponectin, with obesity, plasma lipids and insulin resistance in a Cameroonian population.

**Methods:**

We enrolled 167 men and 309 women aged ≥18 years from the general population in Cameroon. Data were collected on waist circumference (WC), body mass index (BMI), waist-to-hip ratio (WHR), body fat (BF%), fasting blood glucose, plasma lipids, adiponectin, leptin, insulin and homeostasis model for assessment of insulin resistance (HOMA-IR). Pearson’s correlation and multiple stepwise linear regression analyses were used to determine correlates of leptin and adiponectin serum levels.

**Results:**

The prevalence of obesity was higher in women compared to men (*p* < 0.0001), and Central obesity which is more prevalent particularly in women (WC = 42.4 %, WHR = 42.3 %), is almost for 90 % comparable to %BF (42.7 %).

Adiponectin negatively with BMI (*r* = −0.294, *p* < 0.0001), WC (*r* = −0.294, *p* < 0.0001), %BF (*r* = −0.122, *p* = 0.028), WHR (*r* = −0.143, *p* = 0.009), triglycerides (*r* = −0.141, *p* = 0.011), HOMA-IR (*r* = −0.145, *p* = 0.027) and insulin (*r* = −0.130, *p* = 0.048). Leptin positively correlated with BMI (*r* = 0.628), WC (*r* = 0.530), BF% (*r* = 0.720), (all *p* < 0.0001); with DBP (*r* = 0.112, *p* = 0.043), total cholesterol (*r* = 0.324, *p* < 0.0001), LDL-cholesterol (*r* = 0.298, *p* < 0.0001), insulin (*r* = 0.320, *p* < 0.001 and HOMA-IR (*r* = 0.272, *p* < 0.0001).

In multiple stepwise regression analysis, adiponectin was negatively associated with WC (β = −0.38, *p* = 0.001) and BF% (β = 0.33, *p* < 0.0001), while leptin was positively associated with BF% (β = 0.60, *p* < 0.0001), total cholesterol (β = 0.11, *p* = 0.02) and HOMA-IR (β = 0.11, *p* = 0.02). When controlled for gender, HOMA-IR was found significantly associated to adiponectin (β = 0.13, *p* = 0.046), but not BF%, while the association previously found between leptin and HOMA-IR disappeared; BMI and WC were significantly associated with leptin (β = 0.18, *p* = 0.04 & β = 0.19, *p* = 0.02 respectively).

**Conclusion:**

This study, which includes a population who was not receiving potentially confounding medications, confirms the associations previously observed of adiponectin with reduced adiposity especially central adiposity and improved insulin sensitivity. Confirmatory associations were also observed between leptin and obesity, blood lipids and insulin resistance for the first time in an African population. Gender was significant covariate interacting with insulin sensitivity/insulin resistance and obesity indexes associations in this population.

## Background

Africa is experiencing a surge in obesity prevalence and related morbidity and mortality within the context of one of the most rapid epidemiological transition in the world history [1]. Insulin resistance and consequently type 2 diabetes mellitus are major metabolic sequelae of obesity. Adipose tissue has been identified as a link between obesity and insulin resistance [2].

Adipose tissue is now regarded as not just a purely inert body compartment for excess energy storage, but rather as an active endocrine and paracrine organ, secreting a large number of hormones, cytokines and growth factors, collectively called adipokines [3]. Some of them are synthesized exclusively or predominantly by adipocytes (e.g., adiponectin, leptin), while others originate from other sources as well (e.g., resistin, chemerin, proinflammatory cytokines) [4]. Adiponectin and leptin are of particular interest because of their role in the regulation of various physiological processes, including insulin responsiveness, glucose and lipid metabolism, as well as endothelial function, inflammatory response and cytokine signaling [4].

Indeed, there is evidence that adiposity, as measured through body mass index (BMI), and insulin resistance are associated with circulating levels of leptin and adiponectin; leptin has a positive association with adiposity and insulin resistance whereas the converse correlation exists between adiponectin and adiposity and insulin resistance [5–7]. Furthermore, there has been recent interest in the leptin-to-adiponectin ratio (LAR) as a novel predictor of cardio-metabolic and other chronic disease outcomes. This ratio has been shown to be associated with insulin resistance [8, 9], metabolic syndrome [10], carotid intima-media thickness [11], “at-risk phenotype” in young severely obese patients [9], and chronic kidney disease [12], among others.

Adiponectin and leptin circulate in lower concentrations in healthy male compare to female, and is mainly attributed to lower amounts of hexameric High Molecular Weight (HMW) form of adiponectin [13, 14], and to adipose tissue for leptin [15]. Like many other metabolic hormones, adiponectin, especially its HMW form, is regulated by the biological clock showing circadian rhythms with a nocturnal reduction [16]; yet, it’s plasma concentration does not depend on fasting status, show only a limited intra-personal variation over time. On the other hand, leptin shows a circadian variation with a moderate oscillation with their secretion being influenced by regularly eating cycles [17].

Adiponectin and leptin levels vary across ethnic populations. Higher mean levels of leptin and lower mean levels of adiponectin have been reported in African-Americans as compared to subjects of Caucasian ethnicity [18–21]. However, studies that have investigated the role of these adipokines in various diseases among African populations are very scarce. Circulating adiponectin levels were significantly associated with measures of obesity, serum lipids, and insulin resistance in a study on West African population [22]. Circulating Adiponectin was reported to be associated with renal function independent of age and serum lipids in West Africans. According to their findings, the authors suggest that adiponectin may have clinical utility as a biomarker of renal function [23]. A study conducted on urban South African blacks with and without coronary artery disease (CAD) revealed that leptin differentiated between CAD patients with and without metabolic syndrome and determined metabolic syndrome status in women and men [24]. The current study aimed to investigate the potential relationship between leptin and adiponectin, and obesity, blood lipids and insulin resistance in the Cameroonian population. We were particularly interested in determining whose of BMI, waist circumference (WC), waist-to-hip ratio (WHR) and percent body fat (BF %) as surrogates of adiposity, better correlate with leptin and adiponectin plasma levels. Data will contribute to a better understanding of the metabolic effects of these cytokines and their potential as therapeutic targets and predictor of cardio-metabolic outcomes in sub-Saharan Africans.

## Methods

### Study population

This is a cross-sectional study conducted in May 2010 in Douala and Edea, two urban cities of the Littoral region of Cameroon. The study population consisted of individuals of both genders aged ≥ 18 years from the general population, who accepted to participate in the study after an invitation through radio announcements. Pregnant and breast-feeding women, as well as subjects with serious chronic illness, or ongoing or recent (<10 days) acute illness, or those taking any current medication were excluded from the study. After applying the exclusion criteria, 476 participants were included in the study.

### Data collection

For each subject, weight was measured in light clothes with a Seca Scale balance to the nearest 0.1 kg, height with a calibrated stadiometer, waist circumference (WC) at midway between the lowest rib and the iliac crest and hip circumference at the outermost points of the greater trochanters to the nearest 0.5 cm and waist-to-hip ratio (WHR) as waist circumference divided by hip circumference. Body mass index (BMI) was calculated using the Quetelet’s formula as weight (in kg) divided by height (in m^2^). We measured resting blood pressures twice using standardized procedures with the participant in a seated position, and after at least 10 min rest with a validated automated blood pressure measuring device, the Omron HEM-757 (Omron Corporation, Tokyo, Japan). The mean of two measures performed at least three minutes apart was used for all analyses.

Percentage body fat (%BF) was measured by bioelectric impedance analysis using the OMRON BF 302 (OMRON Matsusaka Co., Tokyo, Japan). After 8–12 h overnight fast, blood glucose was measured between 7 and 10 am using the Accu-Chek® Compact Plus glucometer (F. Hoffmann-La Roche AG, Basel, Switzerland) on total fresh capillary blood samples, and venous blood samples were obtained from an antecubital vein. Serum was then separated and stored at −20 °C for lipid measurements and at − 80 °C for further biochemical analysis. Serum lipids were analyzed within one week after the collection. Serum cholesterol (TC) (cholesterol oxidase phenol 4-amino antipyrene peroxidase method), serum triglycerides (TG) (glycerol phosphatase oxidase − phenol4-amino antipyrene peroxidase method), and high-density lipoprotein -cholesterol (HDL-c) (cholesterol oxidase phenol4-amino antipyrene peroxidase method) were measured on a spectrophotometer (UV Mini 1240) using Chronolab kits (Chronolab Systems, Barcelona, Spain). Low-density lipoprotein -cholesterol (LDL-c) was calculated using the Friedwald’s formula [25]. Insulin levels were measured using an electrochemiluminescence immunoassay (Roche Diagnostics, Indianapolis, USA), while serum leptin and adiponectin were measured measured by Radio Immuno Assay (RIA) using Linco Research kits (Linco Research Inc., St Charles, Missouri, USA) with the following characteristics: Adiponectin assay coefficient of variation (CV) ≤ 10 %; detection limit of 2 ng/ml for 100 μl sample size; a linearity range of 500 ng/ml for 100 μl sample size. Leptin assay’s coefficient of variation ≤ 10 % (intra-assay’s CV ≤ 8.3 % and inter assay’s CV ≤ 6.2 %); a detection limit of 0.5 ng/ml for 100 μl sample size; a linearity range of 100 ng/ml for 100 μl sample size. Anthropometrical and biochemical measurements were made once on each participant.

Homeostasis Model Assessment of Insulin Resistance (HOMA-IR) as HOMA − IR = (*Fasting insulin* × *Fasting blood glucose*) ÷ 22.5.

### Definition of anthropomorphic indices of obesity

According to the World Health Organization guidelines, obesity was defined as BMI ≥30 kg/m^2^ [26], WC >94 cm in men and >80 cm in women, WHR ≥0.90 in men and ≥0.85 in women [27]. The BF% cutoffs chosen to define obesity were the values most frequently cited by international scientific literature: BF% ≥ 25 for men and ≥ 35 for women [28].

### Statistical analysis

Data were coded, entered and analyzed using the Statistical Package for Social Science (SPSS) version 20.0 for Windows (IBM Corp. Released 2011. IBM SPSS Statistics for Windows, Version 20.0. Armonk, NY: IBM Corp.). The distribution pattern of the variables was checked. Normally distributed variables are expressed as mean with standard deviation (SD). Skewed variables are reported as median (interquartile range). Skewed variables were log transformed. The independent sample *t*-test was used to compare clinical and biological parameters between men and women. Spearman’s correlation and multiple stepwise linear regression analyses were used to determine correlates of leptin and adiponectin serum levels. Multiple stepwise regression analysis included adiponectin and leptin as dependent variables and on one hand the factors which *p* < 0.05 from the bivariate correlation. These are BMI, WC, BF%, WHR, TG and HOMA-IR for adiponectin, and BMI, WC, BF%, TC, TG, LDL-c and HOMA-IR for leptin. WHR was removed from the final model for adiponectin because it interacts with WC as central obesity index and negative impact on the model. Each model was controlled for age and gender. Statistical tests are two tailed. A *p* value < 0.05 was considered statistically significant.

### Ethical considerations

This study was performed in accordance with the guidelines of the Helsinki Declaration and was approved by the National Ethics Committee for Human Health Research and by the Ministry of Public Health of Cameroon. Written informed consent was obtained from all participants.

## Results

A total of 476 subjects (167 men and 309 women) were enrolled in the study. The median age (interquartile) was 55.0 (23.6) for men and 50.0 (20.0) for women (*p* < 0.05). WHR, SBP and DBP were significantly higher in men (*p* < 0.05 respectively). Triglycerides, LDL-c024), insulin, adiponectin, leptin, and HOMA-IR were significantly higher in women (*p* < 0.05 respectively) (Table 1).

**Table 1:** Clinical and metabolic characteristics of the study population

	Men (*n* = 167)	Women (*n* = 309)	*p* value
Age	55 (23)	50 (20)	<0.0001
Weight (kg)	71.00 (23.00)	70.00 (25.00)	0.984
Height (m)	1.70 (0.20)	1.60 (0.10)	<0.0001
BMI (kg/m^2^)	25.30 (6.30)	28.00 (6.60)	<0.0001
WC (cm)	91.04 ± 12.6	93.58 ± 14.0)	0.091
WHR (cm)	0.91 (0.09)	0.87 (0.09)	<0.0001
BF (%)	22.90 (8.62)	34.00 (10.42)	<0.0001
SBP (mm Hg)	137 (37)	1128 (33)	<0.0001
DBP (mm Hg)	86 (21)	82 (20)	0.006
Blood glucose (mmol/L)	5.0 (1.10)	4.80 (1.15)	0.204
Total Cholesterol (mmol/L)	4.14 (1.07)	4.45 (1.16)	0.05
Triglycerides (mmol/L)	1.20 (0.45)	1.26 (0.35)	0.024
LDL-c (mmol/L)	1.48 (0.97)	1.78 (1.17)	0.031
HDL-c (mmol/L)	2.13 (0.99)	2.14 (0.91)	0.763
Insulin (μU/mL)	2.14 (2.16)	2.73 (2.47)	0.020
HOMA-IR	8.64 (0.25)	11.05 (11.13)	0.031
Adiponectin (μg/mL)	7.65 (5.55)	10.75 (8.90)	0.038
Leptin (ng/mL)	4.80 (6.20)	18.50 (24.40)	<0.0001

WC Values are means (standard deviation); except for WC, values are Median (Interquartile Range)

*WC* waist circumference, *BF%* body fat percentage, *BMI* body mass index, *WHR* waist-hip ratio, *SBP* systolic blood pressure, *DBP* diastolic blood pressure, *LDL-c* low density lipoprotein-cholesterol, *HDL-c* high density lipoprotein-cholesterol, *HOMA-IR* homeostasis model assessment of insulin resistance

### Prevalence of obesity using the different anthropomorphic indexes

**Fig. 1:**
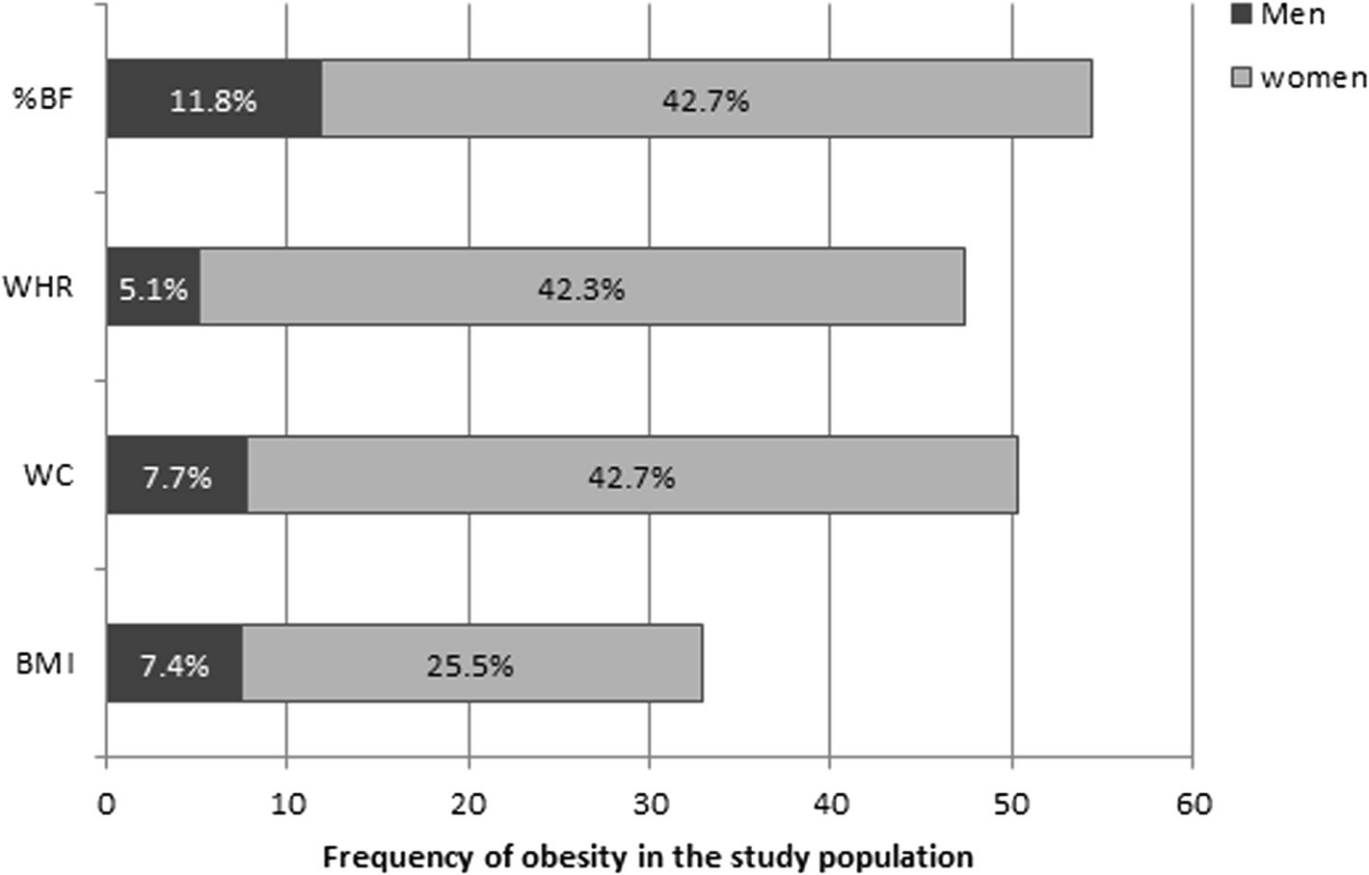
Prevalence rates of obesity within the population using BMI, WC, WHR and BF%

The prevalence of obesity using the different obesity indexes was significantly higher in women than in men (all *p* < 0.001). Men, had a higher prevalence of BF% (11.8 %), BMI and WC showed quasi-equal prevalence rates (7.4 % & 7.7 % respectively); and a low WHR prevalence (5.4 %). In women, WC, WHR and BF% had the higher prevalence than men (42.7 %, 42.3 % and 42.7 % respectively) and lowest prevalence of obesity BMI (425.5 %) (Fig. 1). Central obesity measured with WC and WHR seems to reflect body fat better than general obesity measured with BMI in this population.

### Correlations between adipokines and anthropomorphic indexes of obesity, blood pressure, blood lipids and insulin resistance

As shown in Table 2, adiponectin positively and significantly correlated with age (*r* = 0.200, *p* < 0.0001) and negatively correlated with BMI (*r* = −0.294, *p* < 0.0001), WC (*r* = −0.294, *p* < 0.0001), WHR (*r* = −0.143, *p* = 0.009 and BF% (*r* = −0.122, *p* = 0.028). There was no significant correlation between adiponectin and blood pressure. On the other hand, adiponectin correlated negatively with triglycerides (*r* = −0.141, *p* = 0.011), insulin (*r* = −0.130, *p* = 0.048) and HOMA-IR (*r* = −0.145, *p* = 0.027).

**Table 2:** Spearman’s correlation coefficients of adiponectin and leptin

	Adiponectin		Leptin	
	r	p	r	p
Age	0.200	<0.0001	−0.115	0.038
BMI	−0.294	<0.0001	0.628	<0.0001
WC	−0.294	<0.0001	0.530	<0.0001
WHR	−0.143	0.009	−0.029	0.599
BF%	−0.122	0.028	0.729	<0.0001
SBP	0.009	0.869	0.026	0.643
DBP	−0.009	0.871	0.112	0.043
TC	−0.070	0.207	0.324	<0.0001
TG	−0.141	0.011	0.148	0.007
HDL-c	0.102	0.064	−0.022	0.685
LDL-c	−0.061	0.279	0.298	<0.0001
Insulin	−0.130	0.048	0.320	<0.0001
HOMA-IR	−0.145	0.027	0.273	<0.0001

*WC* waist circumference, *BF%* body fat percentage, *BMI* body mass index, *WHR* waist-hip ratio, *SBP* systolic blood pressure, *DBP* diastolic blood pressure, *TC* total cholesterol, *TG* triglycerides, *LDL-c* low density lipoprotein-cholesterol, *HDL-c* high density lipoprotein-cholesterol, *HOMA-IR* homeostasis model assessment of insulin resistance

There was a significantly positive correlation between leptin and BMI (*r* = 0.628, *p* < 0.0001), WC (*r* = 0.530, *p* < 0.0001) and BF% (*r* = 0.729, *p* < 0.0001). Leptin also positively correlated with DBP (*r* = 0.112, *p* = 0.043), total cholesterol (*r* = 0.324, *p* < 0.0001), LDL-cholesterol (*r* = 0.298, *p* < 0.0001), Triglycerides (*r* = 0.148, *p* = 0.007), insulin (*r* = 0.320, *p* < 0.0001) and HOMA-IR (*r* = 0.273, *p* < 0.0001).

**Fig. 2:**
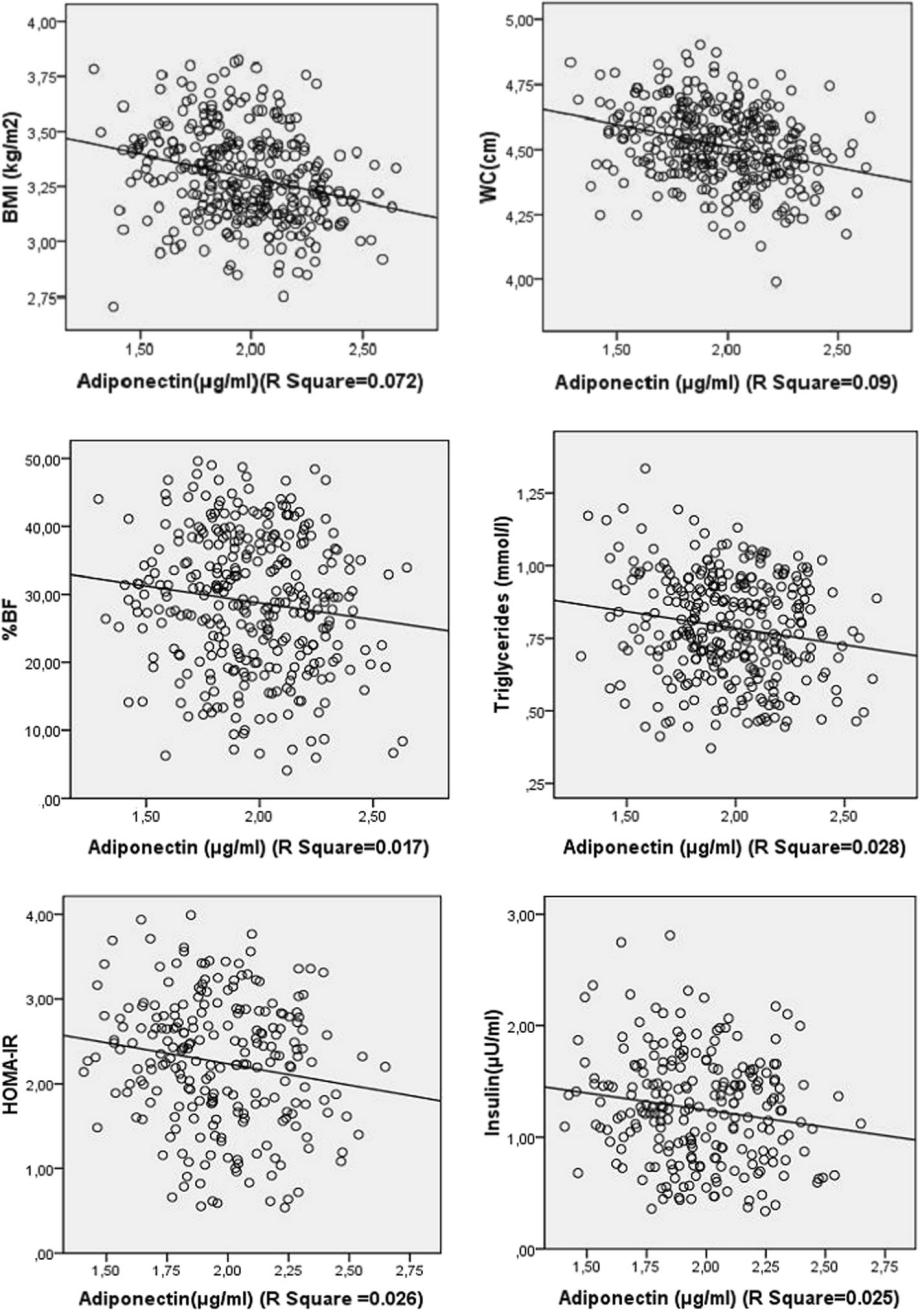
Linear regression presenting the associations of adiponectin with BMI, WC, BF%, triglycerides, insulin and HOMA-IR

In multiple stepwise regression analysis, WC was negatively associated with adiponectin (β = −0.38, *p* < 0.0001, CI: −0.009–−0.0004) and remained unchanged after control for age and gender; a model that explained 26 % of the variance of adiponectin levels. WC is then the index of obesity that better predict adiponectin levels in this population. The positive association between BF% and adiponectin (β = 0.33, *p* < 0.0001, CI: 0.004–0.013) was weakened by age and strongly dependent of gender. HOMA-IR was negatively associated with adiponection when controlled for gender (β = −0.125, *p* = 0.045, CI: −0.081–0.000). That association was significant (p = 0.05) when age was added in the model. Age may impact on the relation between insulin resistance and adiponectin in this population. Age and gender were both significant covariates positively associated with adiponectin (β = 0.24, *p* < 0.0001, CI: 0.002–0.007 & β = 0.29, *p* < 0.0001, CI: 0.09–0.22 respectively) (Table 3, Fig. 2).

**Table 3:** Multiple regression analysis with adiponectin as dependent variable

	No control for age nor gender (R^2^ = 0.19)			Control for age (R^2^ = 0.21)			Control for gender (R^2^ =0.20)			Control for gender and age (R^2^ = 0.26)		
	Beta	*p*	95 % CI	Beta	*p*	95 % CI	Beta	*p*	95 % CI	Beta	*p*	95 % CI
Age				0.17	0.01	0.001–0.010				0.24	<0.0001	0.002–0.007
Sex							0.25	<0.0001	0.06–0.19	0.29	<0.0001	0.09–0.22
BMI	−0.21	0.10	−0.59–0.05	−0.13	0.39	−0.54–0.21	−0.15	0.19	−0.50–0.10	0.06	0.70	−0.30–0.46
WC	−0.38	0.001	−0.01–−0.002	−0.53	<0.0001	−0.18–0.59	−0.32	<0.0001	−0.008–−0.08	−0.37	<0.0001	−0.009–−0.004
BF%	0.33	<0.0001	0.004–0.01	0.21	0.01	0.002–0.01	0.17	0.18	−0.002–0.01	−0.10	0.38	−0.01–0.003
TG	−0.09	0.16	−0.33–0.05	−0.06	0.32	−0.28–0.09	−0.07	0.25	−0.30–0.08	−0.08	0.20	−0.30–0.6
HOMA-IR	−0.11	0.96	−0.08–0.01	−0.10	0.13	−0.07–0.01	−0.13	0.046	−0.08–0.000	−0.12	0.05	−0.08–0.000

*CI* Confidence Interval, *BMI* body mass index, *WC* waist circumference, *BF%* body fat percentage, *TG* triglycerides, *HOMA-IR* homeostasis model assessment of insulin resistance

**Fig. 3:**
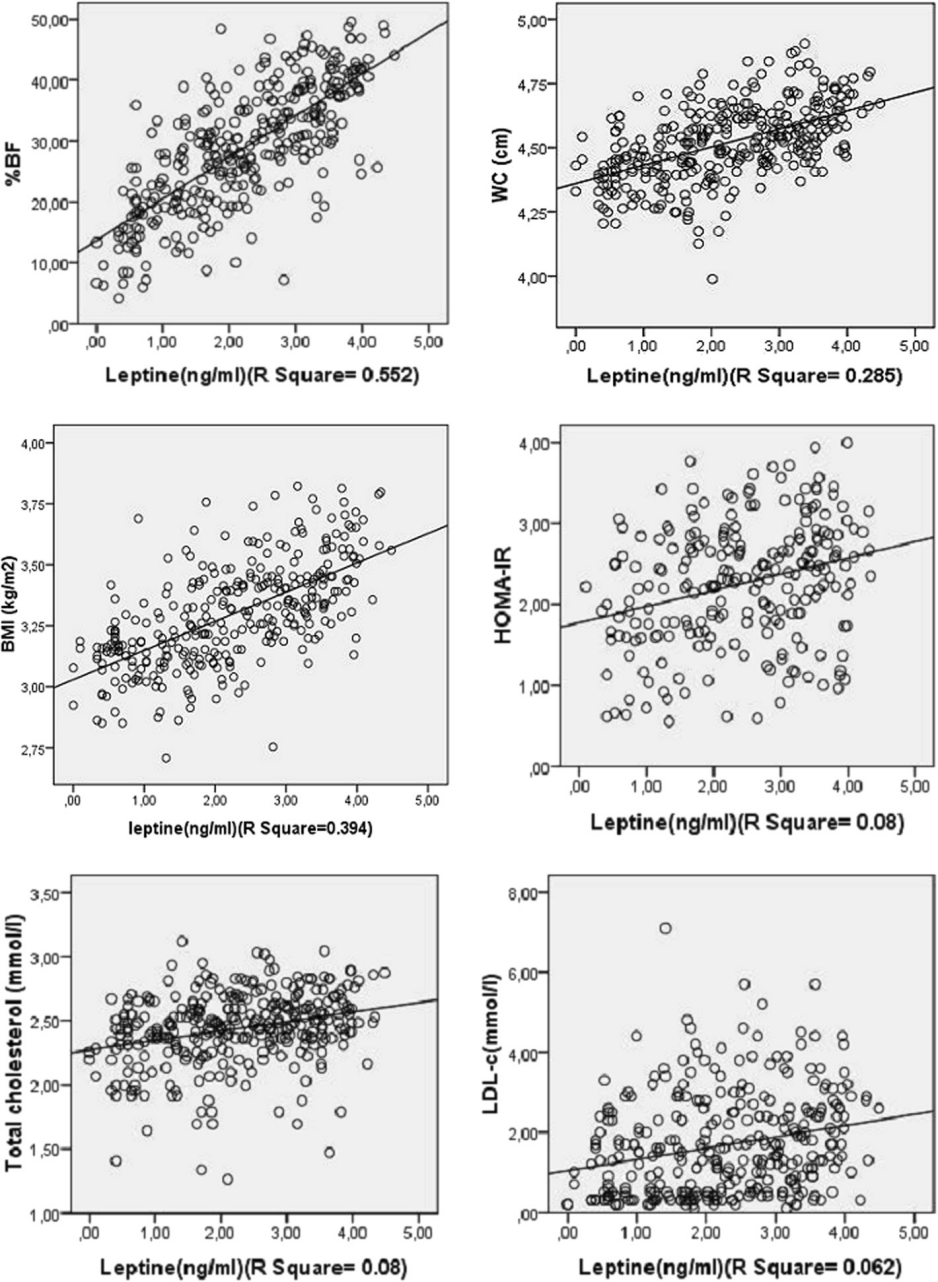
Linear regression presenting the associations of leptin with BMI, WC, %BF, total-cholesterol, LDL-cholesterol and HOMA-IR

Leptin was positively associated with BF% (β = 0.60, *p* < 0.0001, CI: 0.05–0.08), TC (β = 0.11, *p* = 0.02) when controlled for age and gender, a model that explain 68 % of leptin levels. When controlling for gender only, BMI and WC were both positively associated with leptin (*p* = 0.04 & *p* = 0.02 respectively). Age may strongly influence the relation between BMI and leptin. Except for BF%, WC was the obesity index significantly associated to leptin after control for age and gender. The association of HOMA-IR with leptin (β = 0. 11, *p* = 0.02 CI: 0.0–0.29) was dependent of gender, although reinforcement of age. Age and gender were both significant covariates positively associated to leptin (*p* = 0.001, *p* < 0.0001 respectively) (Table 4, Fig. 3).

**Table 4:** Multiple regression analysis with leptin as dependent variable

	No control for age nor gender (R^2^ = 0.60)			Control for age (R^2^ = 0.65)			Control for gender (R^2^ = 0.66)			Control for gender and age (R^2^ = 0.68)		
	Beta	*p*	95 % CI	Beta	*p*	95 % CI	Beta	*p*	95 % CI	Beta	*p*	95 % CI
Age				−0.23	<0.0001	−0.02–−0.01				−0.16	0.001	−0.02–−0.005
Sex							0.35	<0.0001	0.51–1.01	0.24	<0.0001	0.26–0.81
BMI	0.13	0.06	−0.03–0.12	−0.11	0.28	−0.75–0.50	0.18	0.04	0.03–0.09	−0.02	0.99	−0.14–0.12
WC	−0,02	0,81	−0,04–0,01	0.07	0.22	−0.02–0.002	0.19	0.02	0.01–0.03	0.22	0.001	0.06–0.07
BF%	0.60	<0.0001	0.05–0.08	0.75	<0.0001	0.07–0.09	0.29	0.001	0.01–0.05	0.48	<0.0001	0.03–0.07
DBP	−0,01	0,91	−0,62–0,55	−0.04	0.35	−0.29–0.81	0.06	0.19	−0.18–0.92	0.05	0.23	−0.21–0.88
TC	0.11	0.02	0.07–0.84	0.10	0.02	0.06–0.79	0.12	0.01	0.13–0.85	0.12	0.007	0.14–0.85
TG	−0,04	0,35	−0,87–0,31	−0.06	0.14	−0.95–0.14	−0.05	0.22	−0.89–0.21	−0.07	0.09	−0.97–0.08
LDL-c	−0,07	0,21	−0,16–0,04	−0.03	0.53	−0.12–0.06	−0.03	0.56	−0.13–0.07	−0.01	0.91	−0.10–0.09
HOMA-IR	0.11	0.02	0.02–0.29	0.11	0.015	0.03–0.28	0.07	0.12	0.02–0.22	0.09	0.03	−0.01–0.26

*CI* Confidence Interval, *WC* waist circumference, *BF%* body fat percentage, *BMI* body mass index, *TC* total cholesterol, *TG* triglycerides, *LDL-c* low density lipoprotein-cholesterol, *HOMA-IR* homeostasis model assessment of insulin resistance

## Discussion

During the last decade, several studies demonstrated the important role of adiponectin and leptin in the regulation of various physiological processes, including insulin responsiveness, glucose and lipid metabolism, as well as endothelial function, inflammatory response and cytokine signaling [4]. These adipokines have therefore been postulated to be strong predictors of cardiometabolic and other chronic disease outcomes [5–12]. Much more, adiponectin has been identified as a therapeutic target and several strategies have been suggested for increasing its favorable effects [29–31]. This study was designed to explore the relationships of leptin and adiponectin with various anthropometric indexes of obesity, blood lipids and insulin resistance among a Cameroonian population.

We found that plasma adiponectin concentrations are positively correlated to age, and negatively related with BMI, WC, BF%, WHR, triglycerides, insulin levels, and HOMA-IR. In contrast, leptin plasma levels positively correlated with BMI, WC, BF%, DBP, total cholesterol, triglycerides, LDL-cholesterol and insulin levels, and HOMA-IR. The finding of an inverse relationship between BMI, WC and BF% is similar to previous reports in Americans [32], Japanese subjects [33], Pima Indians and Caucasians [34]. A similar association was also found among African-American in the Multi-Ethnic Study of Atherosclerosis (MESA) [35]. In a previous study, we also found that adiponectin was inversely correlated with waist circumference in apparently healthy Cameroonians [36]. This negative correlation between adiponectin and these surrogates of obesity may be due to the fact that adiponectin gene expression is down-regulated in obesity and its related pathology [12, 32]. Furthermore, we found that except for BMI, WC exhibits the stronger negative correlation with adiponectin levels. Yet, the responsibility of subcutaneous or visceral fat in this association is discussed [37, 38]. This result is in accordance with the observation that cultured adipocytes derived from the intra-abdominal fat depot secrete adiponectin more actively than subcutaneous fat derived adipocytes [39]. In this study, WC was the only obesity index significantly associated to adiponectin when controlled for age and gender. This finding confirms the conclusions that WC is the obesity index the better correlate with obesity related diseases [40].

It has been demonstrated that plasma adiponectin concentrations correlate negatively with insulin resistance (or correlate positively with insulin sensitivity) and fasting plasma insulin concentrations [32, 34, 41–44]. Likewise, we found that adiponectin levels are negatively associated with plasma insulin level and HOMA-IR; however, these associations seem to depend on gender in this population. These findings support the notion that adiponectin concentrations could modulate insulin action. Indeed, of the adipokines, adiponectin may be the most biologically active in improving insulin sensitivity. Adiponectin is believed to improve insulin sensitivity by binding to its receptors including AdipoR1 which is expressed primarily in skeletal muscle, and AdipoR2 which is expressed most abundantly in the liver. These receptors mediate the adiponectin-induced increase in glucose uptake in muscle and the decrease in glucose production by liver (hepatic gluconeogenesis), respectively [45, 46]. Because of this important role on insulin sensitivity, adiponectin is currently one of the strongest biochemical predictors of type 2 diabetes mellitus [47]. However, sex hormons can modify the expression and activities of PPARγ and its downstream adipokines [48].

Besides its action as an insulin sensitizer, adiponectin exerts an anti-atherogenic activity by increasing fatty acid uptake and oxidation through the activation of AMP activated protein kinase (AMPK), p38-mitogen-activated protein kinase (MAPK), and peroxisome proliferator-activated receptor gamma (PPARα) pathways [49–52]. We found that plasma adiponectin concentrations are negatively correlated with circulating triglycerides concentrations. These findings are in line with previous studies which reported that serum levels of lipids and lipoproteins (triglycerides, LDL-cholesterol, apolipoprotein A1 and apolipoprotein B) were significantly increased but HDL-C was decreased with decreasing adiponectin levels. It is assumed that the association of adiponectin with lower triglyceride concentrations is secondary to its insulin-sensitizing properties [53].

Leptin has been identified as an important protein involved in the weight regulation system, and the absence of leptin leads to increased appetite and decreased energy expenditure and subsequently obesity [54]. The interaction between leptin and fat mass and obesity-associated (FTO) gene expression is a major mechanistic pathway underlying the role of leptin in the regulation of body-weigh [4]. Interestingly, this study confirms the positive and strong association found between serum leptin levels and percentage body fat. WC and BMI were also found associated to leptin after control for gender. This association of leptin with obesity has been previously demonstrated in a multi-ethnic population including African-Americans [35], as well as in subjects with various obesity-related pathologies such as in type 2 diabetes [44, 55, 56]. Furthermore, we found that leptin plasma concentrations also positively correlated with plasma insulin concentrations and HOMA-IR thus insulin resistance. These findings have been reported in previous studies in type 2 diabetes patients [44, 55, 56]. In fact, hyperinsulinemia and insulin resistance were reported to induce hyperleptinemia through increased adiposity [53, 54]. Beside, the positive correlation between leptin and DBP in this study is in accordance with observational data associating leptin with cardiovascular disease [4], as well as the positive association found with plasma total cholesterol. These associations of leptin with raised blood pressure and pro-atherogenic blood lipids, along with its role in insulin resistance and endothelial dysfunction, in diabetes and hypertension onset, and its pro-inflammatory activity are the major mechanisms through which leptin triggers cardiovascular diseases [4].

Our study has a principal strength that this population was not receiving potentially confounding medications during the investigation. Nevertheless, this study has three main limitations. First, its cross-sectional design prevented us from identifying cause-and-effect associations between leptin and adiponectin, and blood lipids, obesity and insulin resistance. Secondly, we used HOMA-IR to evaluate insulin sensitivity rather than gold-standard methods such as the hyperinsulinemic euglycemic clamp and the frequently sampled-intravenous glucose tolerance test which, however, would have been significantly very difficult to perform in such a population-based study in a resource-limited setting. Despite these shortcomings our study is one the very few to report on the relationship of leptin and adiponectin with lipid profile, anthropomorphic indexes of obesity and insulin sensitivity among subjects of African ancestry. Thirdly, in this study we used a commercially available RIA method (Linco Research Inc.) which measures only total adiponectin, though recent studies suggest that the high molecular weight type of adiponectin is biologically active.

## Conclusions

This study confirms the associations previously observed between adiponectin and leptin, and obesity, blood lipids and insulin resistance. Adiponectin is associated with reduced adiposity, especially abdominal adiposity, improved insulin sensitivity and lower levels of pro-atherogenic blood lipoproteins. In spite of the interactions BF% and HOMA-IR with gender in their association to adiponectin, adiponectin is thus suggested to be a predictor of good metabolic and cardiovascular health in this sub-Saharan African population. Contrariwise, leptin is strongly associated to anthropometric indexes of obesity (BMI, WC, WHR, BF%) and obesity-related adverse effects that trigger cardiovascular disease, including insulin resistance for the first time in an African population, increased blood pressure and pro-atherogenic blood lipids. The associations of HOMA-IR, BMI and WC with leptin were related to gender in this population.

## Ethics approval and consent to participate

The authors attest that the present study was approved by the National Ethics Committee for Human Health Research and by the Ministry of Public Health of Cameroon. Written informed consent was obtained from all participants.

## Abbreviations

AMPK: AMP-activated protein kinase
BF%: percent body fat
BMI: body mass index
HDL-c: high-density lipoprotein –cholesterol
HMW: high molecular weight
HOMA-IR: homeostasis model for assessment of insulin resistance
LDL-c: low-density lipoprotein –cholesterol
PPARα: peroxisome proliferator-activated receptor gamma
TC: total cholesterol
TG: triglycerides
WC: waist circumference (WC)
WHR: waist-to-hip ratio
